# Comparison of Practical Skills Teaching by Near-Peers and Faculty

**DOI:** 10.1097/ACM.0000000000006003

**Published:** 2025-02-27

**Authors:** Roman Hari, Sarah Oppliger, Diana H.J.M. Dolmans, Sören Huwendiek, Renée E. Stalmeijer

**Affiliations:** **R. Hari** was head of education, Institute of Primary Health Care (BIHAM), University of Bern, Bern, Switzerland, at the time of writing and is now dean of education, University of Bern, Bern, Switzerland, and a general practitioner and PhD candidate, School of Health Professions Education, Faculty of Health, Medicine, and Life Sciences, Maastricht University, Maastricht, the Netherlands; ORCID: https://orcid.org/0000-0002-3972-9056.; **S. Oppliger** was a medical student and research assistant, Institute of Primary Health Care (BIHAM), University of Bern, Bern, Switzerland, at the time of writing and is now a resident, Department of Internal Medicine, FMI Hospitals, Interlaken, Switzerland.; **D.H.J.M. Dolmans** is professor of innovative learning arrangements and an educational scientist, School of Health Professions Education, and Department of Educational Development & Research, Faculty of Health, Medicine, and Life Sciences, Maastricht University, Maastricht, the Netherlands; ORCID: https://orcid.org/0000-0002-4802-1156.; **S. Huwendiek** is head and associate professor for medical education, Department for Assessment and Evaluation, Institute for Medical Education, University of Bern, Bern, Switzerland; ORCID: https://orcid.org/0000-0001-6116-9633.; **R.E. Stalmeijer** is associate professor, Department of Educational Development and Research, School of Health Professions Education, Faculty of Health, Medicine, and Life Sciences, Maastricht University, Maastricht, the Netherlands; ORCID: https://orcid.org/0000-0001-8690-5326.

## Abstract

**Purpose:**

Near-peer teaching is a vital teaching resource in most medical schools, but little is known about the comparative benefits of near-peers and faculty teaching or the learning mechanisms that underlie them. This study explored near-peers’ and students’ perceptions of differences between the way near-peers and faculty teach practical skills.

**Method:**

Using qualitative methods, the authors conducted 4 focus groups with near-peers (n = 22) and 4 focus groups with students (n = 26, years 3–6) at the University of Bern, Bern, Switzerland, between September and December 2022. All participants recently participated in near-peer skills training. Vignettes of typical teaching situations guided the focus group discussions. The reflexive thematic analysis was both inductive and deductive; cognitive apprenticeship teaching methods informed the deductive analysis.

**Results:**

Three major areas of difference were identified in near-peers and faculty skills teaching methods: (1) learning climate, (2) teaching orientation, and (3) reaction to identified competence gaps and students’ questions. Near-peers were perceived to establish a safer learning climate than faculty, lowering the threshold to ask questions. Near-peer teaching was oriented toward the formal curriculum and students’ learning needs, resulting in more tailored explanations focused on examination-relevant content. Faculty oriented their teaching toward clinical practice, which helped students transition to clinical practice but could overwhelm novice students. Faculty better stimulated students to think critically about unanswered questions and how to fill their competence gaps.

**Conclusions:**

Skills teaching by near-peers and faculty differed in teaching climate and orientation. Near-peers saw students as learners, focused on the learning climate and on students’ needs. Faculty saw students as future physicians and facilitated the transition from curricular learning to clinical practice. Curricular design should capitalize on the complementary benefits of near-peer and faculty skills instructors and seek to get the best of both worlds.

Near-peer teaching (NPT) is vital to medical schools,^1^ especially in transferring practical skills.^2^ In NPT, one student teaches fellow students, who are less advanced in the same curriculum typically by 1 or more years.^3^ Medical schools employ near-peers as 1:1 substitutes for faculty to reduce the faculty’s teaching burden.^4^ Although NPT is widely acknowledged as a valuable educational tool,^5^ curriculum designers need a better empirical and theoretical understanding of NPT so they can most effectively integrate NPT into medical curricula.

Most research on NPT has focused on documenting outcomes,^2^ and too little research has elucidated the mechanisms of learning in NPT.^6^ In existing research, the most frequently described mechanisms are cognitive and social congruence.^3,7^ Cognitive congruence argues that near-peers and learners structure knowledge similarly, so near-peers can offer effective assistance^8^; participants’ perceptions of NPT align with this view.^9^ Social congruence argues that the proximity of near-peers and their similar social roles foster a safe learning climate for students^3,10^ where they are comfortable self-disclosing and correcting their errors.^11,12^ However, we do not know whether or how NPT embodies these theories in its various teaching methods.

Teaching methods can be described and improved^13^ through cognitive apprenticeship (CA),^14^ which helps instructors make their “thinking” visible via modeling, coaching, scaffolding, articulation, reflection, and exploration.^14^ Clinical teaching does this in a 3-stage process^15^: (1) the instructor starts the session by creating a safe learning climate and then models the task, (2) the instructor coaches the student to perform the task, each time with less supervision (scaffolding), and (3) the instructor uses articulation and exploration to stimulate self-directed learning. Near-peers and faculty use CA methods to a similar degree, but near-peers spend more time creating a safe learning experience.^16^

This study elucidated and compared the methods near-peers and faculty used to teach practical clinical skills to small groups in each of the 3 phases of CA, so we could identify and capitalize on the strengths of each type of instructor. We aimed to answer the following research questions from the perspective of near-peers and students: How do near-peers and faculty differ in their use of teaching methods in practical skills teaching? How do these differences affect students’ perceived learning? Why do near-peers and faculty use teaching methods differently?

## Method

### Study design

This qualitative study followed a constructivist paradigm. Through focus groups (FGs), we explored students’ and near-peers’ perceptions of near-peer and faculty skills teaching. Guided by reflexive thematic analysis,^17^ our analysis was both inductive and deductive, with the CA teaching methods informing the deductive analysis. All participants provided written informed consent before the start of the interviews. The Cantonal Ethics Committee of Bern, Switzerland, exempted this study from review.

### Setting

From September to December 2022, we collected data at the University of Bern, Bern, Switzerland, where NPT is integrated into all 6 years of the curriculum. Practical skills sessions cover clinical examination and history taking, cannulation, basic life support, and ultrasonography training. The university pays near-peers by the hour and offers a range of training for near-peers, from informal verbal instruction to half-day structured sessions.

### Participants

Our purposive sampling included 2 populations: (1) students who had been recently taught practical skills by near-peers and faculty (students) and (2) medical students who had recently been near-peers (near-peers). In this article, the term *faculty* refers to physicians experienced in teaching, and the term *instructors* is used if both near-peers and faculty are meant. We focused on the hands-on clinical skills required to diagnose and treat patients (practical skills)^2^ because these skills are what near-peers most commonly teach.^5^ Student FGs included participants from the same year to encourage open discussion. Participation was advertised through lectures, a student forum, and a WhatsApp group that is accessible to approximately 320 students per year. Each year, 5 to 8 students applied, which is an ideal size for student FGs.^18^ We recruited near-peers from each skills course based on whether they had taught within the last 2 months: 8 to 25 were eligible per course (see Table 1 for student and near-peer demographic data). Mean age and gender were similar between groups; 13 students (50%) had been near-peers themselves. Participants in the FG received 30 CHF (approximately US $33) each.

**Table 1:** Order and Participants of FGs That Explored Student and Near-Peer Perception of Practical Skills Teaching by Near-Peers and Faculty at the University of Bern, Bern, Switzerland, September to December 2022

FG no.	Participants	Most recent peer-led course^a^	No. of participants	Typical course task	Study year(s)	Taught as near-peer, no. (%)	Teaching experience, mean (range) (y)	Age, mean (range) (y)	Gender, M/F
FG1	Student	Ultrasonography	6	Perform ultrasonography of abdominal organ on fellow student	5	2 (33)	NA	23.8 (22–27)	6/0
FG2	Near-peer	Ultrasonography	7	Perform ultrasonography of abdominal organ on fellow student	5, 6	7 (100)	3.4 (3–4)	23.9 (22–28)	5/2
FG3	Student	Clinical examination	8	Perform systematic examination (e.g., lung) on SP	3	3 (38)	NA	22.0 (21–26)	6/2
FG4	Near-peer	Clinical examination	5	Perform systematic examination (e.g., lung) on SP	4, 5, 6	5 (100)	2.8 (1–5)	24.0 (22–25)	2/3
FG5	Student	Ultrasonography	7	Perform ultrasonography of abdominal organ on fellow student	4	4 (57)	NA	24.7 (22–31)	4/3
FG6	Near-peer	Ultrasonography	5	Perform ultrasonography of abdominal organ on fellow student	4	5 (100)	2.2 (2–3)	23.2 (22–24)	4/1
FG7	Near-peer	Basic life support	5	Perform CPR on mannequin	5, 6	5 (100)	4.4 (2–6)	25.2 (24–26)	3/2
FG8	Student	Various	5	Various	6	4 (83)	NA	25.2 (24–26)	3/2

Abbreviations: CPR, cardiopulmonary resuscitation; FG, focus group; NA, not applicable; SP, standardized patient.

^a^Attended (students) or taught (near-peers).

### Data collection

We held 8 FGs—4 student (n = 26, years 3–6) and 4 near-peer (n = 22) groups; each lasted approximately 90 minutes. We chose to use FGs to gain deeper insights from interactive discussions.^18^ Students and near-peers participated in different FGs to avoid power differentials within the group (see Table 1 for FG order and participant information). We held the FGs in a university meeting room and provided refreshments. S.O. moderated the near-peer FG, and R.H., who employs near-peers, observed. R.H. moderated the student FG and S.O. observed. Both took extensive field notes that were discussed after each session and later reviewed with the research team.

We conducted 2 pilot interviews to refine our preliminary interview guide before we collected data and found that pilot participants struggled to describe teaching methods in detail without additional support. We thus drew on our own teaching experience to write vignettes that depicted typical instructor actions in small-group settings for each phase of CA clinical teaching.^15^ Participants were given the vignettes during the FGs, and the moderator used them to prompt discussion. The FG discussions first focused on differences in teaching methods, comparing students’ and near-peers’ perceptions. After 5 FGs, we updated the vignettes to include the identified preliminary differences, prompting discussions on the reasons (FG6 and FG7, near-peer) and effects (FG8, final-year students) of the differences. See Supplemental Digital Appendix 1 (at http://links.lww.com/ACADMED/B679) for the interview guide and versions of the vignettes.

### Data analysis

**Figure 1:**
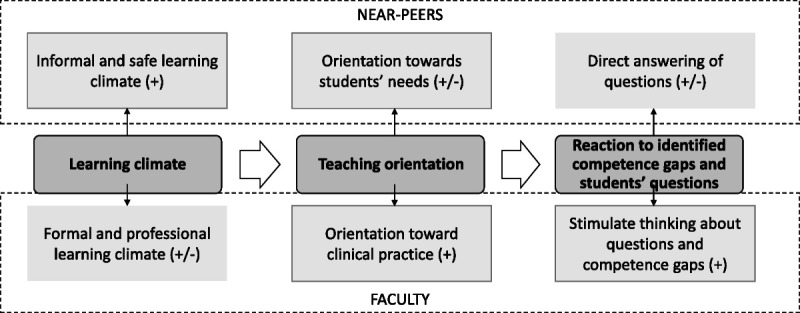
Areas of difference between near-peer and faculty skills teaching (dark gray boxes) perceived by students (n = 26) and near-peers (n = 22) in a qualitative study at University of Bern, Bern, Switzerland, performed between September and December 2022. Typical teaching methods of near-peers (upper dashed box) and faculty (lower dashed box) and their perceived effects on learning are shown. Plus sign indicates positive effect; plus/minus sign, ambiguous effect.

All FGs were audio-recorded, transcribed verbatim, and analyzed through reflexive thematic analysis.^17^ Three researchers (R.H., S.O., R.E.S.) conducted open, inductive coding of the first FG, assigning descriptive codes to significant text segments and memos to capture reflections and highlight key quotes. S.O., R.H., and R.E.S. coded FG1; S.O. and R.H. coded FG2 and FG3; and S.O. coded FG4 to FG8 alone. The research team discussed the codes and iteratively refined and regrouped them under overarching themes,^19^ using both inductive and deductive approaches. Graphical representations of the themes were created to help refine key themes (Figure 1). The resulting themes were reviewed through the lens of CA. We illustrated key findings with quotations from participants (translated into English). MAXQDA, version 2022, release 22.4.1 (VERBI Software GmbH, Berlin, Germany) was used for transcription and coding.

### Reflexivity

The study group comprised 5 researchers. R.H. is a general practitioner pursuing a PhD in health professions education. During data collection, R.H. worked at an institute that organizes near-peer skills teaching. S.O. was a medical student and former near-peer in the same curriculum as the study participant. D.H.J.M.D., S.H., and R.E.S., experienced educational researchers without direct ties to NPT at the University of Bern, provided an outside view so our findings would be transferable.

## Results

We identified 3 major areas of difference in near-peers and faculty skills teaching methods: (1) learning climate, (2) teaching orientation, and (3) reaction to identified competence gaps and students’ questions. Figure 1 shows typical near-peer and faculty teaching methods related to these 3 areas.

Some methods were thought to positively affect learning (e.g., near-peer learning climate and the way faculty stimulated thinking about open, unanswered questions). Other methods were thought to have ambiguous effects. Below, we describe the 3 main themes in detail, contrasting typical near-peer and faculty methods and reporting their perceived effects on student learning. Each section concludes with near-peer and student explanations for the perceived differences. Table 2 provides representative quotations relating to the 3 main themes. Near-peers and faculty often taught skills similarly (e.g., type of teaching methods, strong motivation for skills training), but here we highlight the differences to answer our research questions.

**Table 2:** Differences in Near-Peer and Faculty Teaching Methods of Practical Skills, Perceived by Near-Peers and Students at the University of Bern, Bern, Switzerland, September to December 2022^a^

Theme	Representative quotations
**Learning climate**	
Near-peers: informal and safe learning climate (+)	• “People certainly dare to ask a lot more. Like, if there is someone with 20 years of experience, then you just accept it and think: ‘Ah, I have to look that up’ and with the peers, it’s more like: ‘Ah, do you know why that is?’ or ‘Would it also work like this?’ and they say: ‘No, look,’ and then the reason comes and then it’s ‘Ah, okay, good. Ticked off, in the drawer, learned, done.’” (FG 1, P1, student)
Faculty: formal and professional learning climate (+/−)	• “The climate of the pressure there [in this faculty-led course], together with this fear that one could be exposed, … so that I have just not asked questions where I would have wanted to ask.” (FG8, P48, student) • “I have to be like almost 150% sure I have the right answer until I open my mouth.” (FG1, P3, student) • “If you have a couple of clowns in the group … it would be good to have someone who is serious enough to say: ‘Hey, people, let’s pull ourselves together for a moment.’” (FG1, P1, student).
**Teaching orientation**	
Near-peers: orientation toward students’ needs (+/−)	• “I think it’s really cool to have peer tutors [near-peers] who are close to our own progress because they know exactly where their difficulties were at the beginning…. ‘Ah hey, you have to pay attention to that, that’s rather difficult at the beginning, um, try to keep it that way.’“(FG1, P1, student) • “Near-peers are focused on what is required in the exams, what is required in our studies. But whether a practicing doctor actually needs this afterward is sometimes a completely different reality.” (FG1, P4, student) • “With the exam, you always get to them for sure. [laughter] ‘Attention, it is relevant to the exam’ [laughter].” (FG7, P39, near-peer)
Faculty: orientation toward clinical practice (+)	• “The question was somehow ‘why do you test the rebound tenderness?’ And then [it was] compared with clinical examples, so that you don’t just learn it by heart, but really know afterwards why you are asking this. Why is the answer important? And somehow that helps me, it has helped me a lot to better understand the history taking.” (FG3, P14, student) • “[In the clinic] it just ‘matters’ a little bit more and ‘good’ is simply the standard.” (FG6, P34, near-peer) • “They have much more knowledge to share. And then it is perhaps also more difficult to make a selection, because the temptation is great to add this and that…. Then you might quickly spend another 10 minutes explaining something, even though that is not really the topic.” (FG2, P7, near-peer)
**Reaction to identified competence gaps and students’ questions^b^**	
Near-peers: direct answering of questions (+/−)	• “[With near-peers,] if you don’t answer within three seconds, they’ll come up with an explanation. Which I’m sure is well-intentioned, but you don’t have time to sort it out and think about it.” (FG3, P14, student)
Faculty: stimulate thinking about questions and competence gaps (+)	• “If, for example, you didn’t know something in the prep course, then it’s more like yes, ‘look it up next week and see if you can find it out,’ and then the student [the near-peer] rather just says how it is. And like—fills in the gap.” (FG3, P19, student)

^a^Left column depicts typical near-peer and faculty teaching methods and perceived effects on learning (with + indicating positive effect and +/− indicating ambiguous effect); right column demonstrates these teaching methods using representative quotations from each participant (P).

^b^How near-peers and faculty react if students ask questions or reveal competency gaps.

### Learning climate

**NPT fostered an informal, safe climate for learning, so students were more comfortable asking questions.** Near-peers and students described the learning climate in NPT as less formal and safer, which encouraged students to request clarification and ask questions. At the same time, near-peers felt their social proximity to students sometimes made it harder to maintain discipline throughout the course. This sentiment was echoed by students, who described situations in which they wished for a more professional climate focused on learning.

**Faculty provided a more formal, professional learning climate that increased discipline during courses.** Students reported a high variability among faculty regarding the learning climate. Usually, students appreciated the “professional setting” (FG1, participant [P] 3, student) of faculty teaching, which established a climate of disciplined learning, but this formality sometimes made students hesitate to ask questions. They wanted to give faculty the correct answer, perceiving them as having “natural authority” (FG4, P25, near-peer) derived from their standing and experience.

Among the reasons for differences in learning climate was the social proximity of near-peers, for example, smaller “age difference” (FG7, P41, near-peer) or similar “reality of life” (FG1, P4, student). Students enjoyed this proximity when near-peers showed empathy, remembered their names, or made jokes, creating a nonthreatening, informal climate. In contrast, “hierarchy” (FG1, P4, student) and the “clear distribution of roles” (FG1, P3, student) were factors in making them feel that faculty teaching created a professional learning climate.

### Orientation of teaching

**Near-peers’ orientation toward students’ needs resulted in appropriate level of difficulty and examination-focused learning.** Near-peers were perceived as orienting their teaching more strongly to meet students’ needs. Near-peers involved students more actively in defining lesson content, difficulty, and teaching methods:

Right from the start, [the near-peer] asked what we found difficult, what we still wanted to look at. And also, during the procedure, he kept asking: “Ah, do you want to do this technique again or something else?” … he was a lot, like: “What do you want to do, what is important to you?” (FG1, P4, student)

Students felt near-peers introduced new topics “more slowly” (FG5, P27, student) and used fewer “technical terms” (FG7, P39, near-peer) than faculty. Near-peers anticipated learning difficulties and targeted tips and feedback, providing mnemonics and learning resources.

Students thought it was harder for faculty to diagnose learning problems; faculty had forgotten what it was like to experience typical “struggles from the beginning” (FG1, P1, student). Instead, they had automated many procedural processes, so it was harder for them to divide a task into smaller parts. Near-peers knew the learning materials and learning goals of the courses better than faculty because they had recent personal experience. Near-peers also adhered more closely to the course structure, which was helpful.

With the peer tutors [near-peers] it is always very clear, ah okay, these are the learning goals, you have to be able to do this, this leads to these exams or to the OSCEs [objective structured clinical examinations] and there you have to apply it in such and such a way so that it is correct. And that usually helps a lot for the learning effect. (FG1, P4, student)

This quotation also illustrates that NPT is oriented to the examination because near-peers both empathized with students’ fear of the examination and used it to motivate students to learn. Students new to a topic or under pressure to perform concurrent learning tasks felt better prepared for examinations after near-peer–led lessons and were less stressed, but both near-peers and students worried that examination-oriented learning might not be sustainable or adequately prepare students for clinical practice.

**Faculty orientation toward clinical practices motivated and anchored learning but could overwhelm novice learners.** Faculty often focused on educating students in skills needed for patient care but sometimes neglected or questioned learning goals less directly relevant to clinical practice. Faculty emphasized clinical outcomes, such as correct diagnosis, over teaching necessary skills:

What is relevant for him [the faculty] is to show the kidney or to show the vena cava correctly and not “How do I get there?” or “How do I guide the transducer?” (FG5, P33, student)

Faculty often gave outcome-focused feedback, oriented toward a high standard of care in clinical practice, which motivated and helped students but also increased pressure on learners. In contrast, near-peers valued smaller learning steps, even if the result was not yet perfect.

Faculty had the clinical experience to situate learning in clinical practice. By relating content to clinical practice, faculty helped students better contextualize their learning. Students appreciated practical tips derived from clinical practice, but novice students especially felt too much clinical context could be distracting or overwhelming.

Exploring the reasons for these differences in teaching orientation, near-peers often mentioned their previous personal learning experience as guidance for their teaching. In contrast, faculty teaching tended to foreground patient care as the benchmark for education.

### Dealing with questions and competence gaps

**The empathy of near-peers may lead them to give direct answers to student questions, without asking leading questions to help the student arrive at the answer.** Often, when students asked questions, near-peers struggled to encourage independent thinking. Although students appreciated near-peers’ empathy and willingness to help, they did not appreciate these early interventions.

**Faculty stimulated students to think about questions and fill competence gaps, making learning more sustainable.** Students thought faculty gave them more time to find their own solutions and sometimes to develop learning goals for further learning. Students perceived that having to come up with their solutions was helpful and that their own answers were “things that are much more likely to remember because you have tried to think for yourself” (FG3, P14, student). Faculty also tended to react differently when they identified competence gaps in students:

For example, in percussion, if they [students] tap off mark, I always say “that's a matter of exercise. Practice it a bit yourself at home. It's not a problem if it doesn't work the first or second time” … the doctors did relatively little of that with us. So, it was made quite clear that actually, you have to be able to do it. (FG4, P23, near-peer)

Near-peers and students felt there were good and bad outcomes of emphasizing competence gaps: it could either encourage learning or pressure students until they became demotivated. Participants attributed most of these differences to the social proximity of near-peers: “they feel sorry for you and don’t want to stress you out and don’t want to make you feel, like: well, you should know that!” (FG2, P14, student).

## Discussion

Near-peer teaching is widely used in medical education as a substitute for faculty teaching^1^ but could be further capitalized on if the teaching methods and learning mechanisms of NPT were better understood.^6^ This study sought to describe differences between near-peer and faculty skills teaching as perceived by students and near-peers. It explored the reasons for and experienced effects of these differences. We identified 3 main differences in near-peer and faculty teaching: learning climate, teaching orientation, and handling of student questions and competence gaps. These differing approaches had mainly positive but sometimes ambiguous effects on students’ perceived learning. The discussion starts with the fundamental difference in teaching orientation of near-peers and faculty. We then reflect on the ambiguous effects of social congruence on perceived learning in its effect on the learning climate and the handling of student questions and competence gaps.

### Difference in teaching orientation

According to participants, the key difference between near-peer and faculty teaching was their orientation. Near-peers orientated their teaching to learning goals defined by the curriculum and examination. Other studies also found near-peers can be more examination oriented,^20^ but our study adds insight into the cognitive congruences at play^7^ and how this translates into helpful teaching methods. Because near-peers adapt their pace and language to meet students’ needs, they give more targeted tips and feedback. In contrast, faculty are more oriented toward patient care and use their clinical experience to embed skills in real-world clinical contexts; they offer authentic learning experiences even when skills training sessions do not involve patients,^21^ which may help learners transition to become members of a community of practice.^21^

Our participants attributed differences in orientation to the recent personal learning experiences of near-peers and the experience faculty have accrued while caring for patients. We argue that these different exposures are the root of instructors’ differing perspectives. Near-peers see students as learners based on their own recent memories of learning skills. Faculty see students as future physicians, and so they focus on developing the competencies necessary to perform a skill as a member of a health care team; they link learning to clinical practice and real patients. Near-peers and faculty together emphasize the distinct roles of medical students.^22^ The faculty’s approach aligns more closely with constructivist learning principles: learners are motivated by authentic and holistic learning tasks.^23^ Younger near-peers may not realize that preparing for an examination does not always prepare students for clinical practice. As near-peers gain more clinical experience and eventually become faculty members themselves, their teaching orientation may gradually shift from empathy with students struggling to learn (near-peers) to improving patient outcomes (faculty). Researchers should trace the evolution of the role of near-peers through the curriculum as they move from student to practicing clinician and faculty.

### Ambiguous effects of social congruence

Participants described near-peers as “closer” to learners because of social proximity and similar life situations, which aligns with findings of social congruence in the literature.^7,9^ We identified 2 differences related to social congruence in NPT methods. The first is that social congruence allowed near-peers to create a more informal and safe learning climate, lowering the barrier to asking questions. However, near-peers’ high social congruence makes it more difficult for them to lead discussions and persist on learning goals. Near-peers answered questions too quickly and downplayed student competence gaps, depriving students of opportunities to think critically and learn independently. Students attributed this behavior to near-peer empathy for students who might feel uncomfortable answering hard questions. These results match findings from a study of a longitudinal train-the-teachers course, where near-peers struggled to help students find their own answers to challenging questions.^24^

Faculty teaching was described as more formal, creating a disciplined and professional learning climate in which students risked discomfort when they asked questions. Other studies that compared faculty with near-peers in skills teaching^25^ and nonskills learning^10^ returned similar results. (We do not know whether faculty status or their behavior contributes more to creating a formal atmosphere.) Faculty were better at sustaining the tension that arose when students had competency gaps, and they helped students to formulate their own learning goals. To ensure the success of NPT, we need to study the mechanisms that maintain a balance between maintaining safe learning and sustaining the necessary tension for fostering critical thinking and then devise methods for training near-peers to do so.

### Implications for practice

Our study suggests that near-peers and faculty have complementary strengths. Medical schools should integrate NPT into their curricula as a deliberate educational design choice. Near-peers can help establish a nonthreatening learning climate^26^ where students can safely learn new clinical skills.^27^ The strength of near-peers lies in their compassion and insight into student learning processes, whereas faculty are better suited to transferring learning to clinical practice and supporting self-directed learning.^28^ Structured train-the-teacher programs^29^ should help each group understand its strengths and weaknesses and develop their competence as instructors.^30^ Faculty and near-peers should learn from one another during exchanges that allow them to share their perspectives on learning. Near-peers should be trained to handle student questions constructively to promote active thinking and self-directed learning.

### Strengths

A main strength of this study is its solid conceptual foundation in CA in planning the study, designing the vignettes, and structuring the results. Linking CA as a framework for instructional design with theories on the psychology of NPT allowed us to gain a deeper understanding of NPT and translate the results into suggestions for instructional design. Gradually shifting the direction of the FGs from identifying differences to exploring effects (students) and reasons (near-peers) through adapted vignettes provided a holistic perspective on near-peer skills teaching with efficient use of resources.

### Limitations

This study also has some limitations. First, teacher and student performance measures are lacking to assess the impact of differences in teaching methods. Second, we did not include faculty members as participants in this study. Future studies should explore the faculty perspective, especially their thoughts on why near-peers and faculty use teaching methods differently. Third, although 50% of the members of the student FGs had been near-peers and S.O. had also previously worked as a near-peer, our results may have been biased toward the perceptions of near-peers. We suggest future researchers test the effect of NPT on student perceptions and stratify their analysis by near-peer–naive students. Fourth, because our study focused on perceptions of skills training, it was not designed to capture differences in other essential teaching activities (e.g., handling group discussions or guiding clinical reasoning).

### Conclusions

Skills courses taught by near-peer and faculty differed in 2 key ways: teaching climate and orientation. Near-peers see students as learners, help introduce students to new topics, and guide them to gain the knowledge they need to pass examinations. Faculty see students as future physicians and encourage them to be continuous, self-directed learners, supporting them in their transition from curricular learning to clinical practice. To best implement near-peer skills teaching in their curricula, medical schools should aim for the best of both worlds. They should capitalize on the strengths of near-peers, provide training to near-peers to improve their ability to ask leading questions and encourage self-direct learning, and continue to conduct educational research that explores the effects of these adaptations.
